# Starter Kit for Geotagging and Geovisualization in Health Care: Resource Paper

**DOI:** 10.2196/23379

**Published:** 2020-12-24

**Authors:** Quan Do, David Marc, Marat Plotkin, Brian Pickering, Vitaly Herasevich

**Affiliations:** 1 Mayo Clinic Rochester, MN United States; 2 College of St Scholastica Duluth, MN United States; 3 Emory University Atlanta, GA United States

**Keywords:** geographic mapping, medicalGIS guidelines, information storage and retrieval, mapping, geotagging, data visualization, population, public health

## Abstract

**Background:**

Geotagging is the process of attaching geospatial tags to various media data types. In health care, the goal of geotagging is to gain a better understanding of health-related questions applied to populations. Although there has been a prevalence of geographic information in public health, in order to effectively use and expand geotagging across health care there is a requirement to understand other factors such as the disposition, standardization, data sources, technologies, and limitations.

**Objective:**

The objective of this document is to serve as a resource for new researchers in the field. This report aims to be comprehensive but easy for beginners to understand and adopt in practice. The optimal geocodes, their sources, and a rationale for use are suggested. Geotagging’s issues and limitations are also discussed.

**Methods:**

A comprehensive review of technical instructions and articles was conducted to evaluate guidelines for geotagging, and online resources were curated to support the implementation of geotagging practices. Summary tables were developed to describe the available geotagging resources (free and for fee) that can be leveraged by researchers and quality improvement personnel to effectively perform geospatial analyses primarily targeting US health care.

**Results:**

This paper demonstrated steps to develop an initial geotagging and geovisualization project with clear structure and instructions. The geotagging resources were summarized. These resources are essential for geotagging health care projects. The discussion section provides better understanding of geotagging’s limitations and suggests suitable way to approach it.

**Conclusions:**

We explain how geotagging can be leveraged in health care and offer the necessary initial resources to obtain geocodes, adjustment data, and health-related measures. The resources outlined in this paper can support an individual and/or organization in initiating a geotagging health care project.

## Introduction

### Background

Medical geographic information systems (MedicalGIS), in general, and geotagging, in particular, have been employed in epidemiological research and population health practice to understand the correlation between locational information and health or health care. The instruments supporting MedicalGIS include GIS, big data, artificial intelligence such as agent-based modeling, cellular automata, disease surveillance, and analytical models. The uses of this technology in public health include but are not limited to estimating possible access to care, exploring the population distributions of health (eg, neighborhood socioeconomic conditions and population density), determining spatial allocations of diseases, and identifying the proximity to environmental health hazards. While GIS is a type of system that combines multilayers of geographic data to smoothly facilitate information and knowledge about data that is related to the location [1], geotagging is the process of attaching geographical identification tags to various media such as videos, photos, and websites [2]. These geospatial tags carry information such as longitude, latitude, name of a location, and distance. Geotagging carries both pros (eg, improving safety on the trail, targeting marketing, providing more context) and cons (eg, privacy and security, overtourism) [2].

The application of geotagging in health care traces back before the modern medicine era and the development of computers. During the 1854 cholera outbreak in London, Dr John Snow traced the cause of this pandemic by identifying that clusters of the disease plotted on a map near a city water pump [3]. This early example of using location with other data sources exemplifies the potential opportunities that geotagging can serve in health care. Today, the field that focuses on the use of geographic information and methods to study disease and health is known as medical geography or MedicalGIS. MedicalGIS began with early maps to understand the spread of disease and now includes the application of GIS to visualize and analyze trends related to the treatment, prevention, progression, and impact of disease [4].

With the evolution of medical geography, there has been greater national attention on creating a health care system to support the analysis of geographic location. An example of a national effort to adopt geotagging is from the US government’s report Healthy People 2010, [5] which set a goal of geotagging 100% of all national, state, and local health datasets. Unfortunately, in the final report for this initiative, there was no measurable improvement in geotagging major health systems [5]. Despite the limitations in a recent national endeavor to adopt geotagging in health care data, there are clear examples where geotagging has shown promise.

Today, geotagging is used in many industries including retail, government, insurance, technology, and health care. In health care, the goal of geotagging is to gain better understanding of health-related questions applied to populations. Traditionally, there has been a prevalence of geographic information in public health. However, in order to effectively use and expand geotagging in health care, there is a requirement to understand other factors such as disposition, standardization, data sources, and limitations. It is important to have knowledge of the accompanying software to make use of the data for application. This paper will consolidate basic knowledge to assist in the initiation of a geotagging analysis in health care. The paper is designed as a resource document by suggesting the optimal geocodes, their sources, and a rationale for their use.

### Geotagging in Public Health

Geotagging has been used for multiple purposes in health care. Some of the main benefits of geotagging are (1) being a better tool for health care data’s visualization [6,7], (2) supporting time domain data analysis [8], (3) facilitating cross-analysis of multiple data types [9-11], and (4) enabling automatic data analysis [12-14].

Geotagging plays a crucial role in the visualization of geographically related health care data. Geotagging facilitates not only the analysis of this type of data, but also policy decision making [6], planning of new research, and data acquisition activities [7]. In a recent research study, geographical data visualization was used to explore the distribution and potential level of fluoride and arsenic in drinking water in Mexico in order to approximate the corresponding health burden for proper public health policies [6]. The study confirmed the association between fluoride contamination and aridity, together with the variation of fluoride concentrations in arid and humid states [6]. In China, GPS and GIS were used to locate and identify the distribution of malaria. Mobile terminal data acquisition was developed to collect such data for the purpose of malaria prevention and control [7].

Geotagging also helped to analyze geographically related time-varying data. It can be employed to evaluate the effectiveness of interventions (eg, evaluating the effectiveness of social distancing for individual neighborhoods in order to provide a more reasonable distribution of resources to assist high-risk populations) [15], predict trends in population health and health care (eg, using Twitter data to advance Zika virus surveillance) [16], determine the urgency level of interventions (eg, generating acute ischemic stroke alert or detecting COVID-19 symptoms, testing access, and recovery) [17,18], and identify areas that require further investigation (eg, worsening of the population health due to environmental pollution) [19]. Researchers of a study conducted in Japan in 2018 obtained geographical movement of physicians to determine the most favorable location and the median distance of geographical movement of female and male groups of health care providers [8]. The authors concluded that physicians preferred to work in urban areas but there was a higher rate of female physicians who had their first preference of working location as urban areas. Also, the overall distance of geographical movement among female providers was lower compared with male physicians. Finally, the greatest moving distance occurred between their second and fourth years after obtaining their medical license [8].

Data integration is essential in this era of big data. Geotagging contributes an important role in supporting cross-analysis of multiple data types such as environmental data, industrial data, demographic data, and sociological data. GIS has been used to determine potential patients and target locations for marketing purposes at hospitals [9]. Remote sensing data was integrated with geotagging to track the water quality of the Ismailia Canal in Egypt [10]. Transportation data generated and collected from different types of sensors was also used in an artificial information (AI)-based deep learning system to track patterns in the mobility of residents for developing better transportation models of smart cities [11].

When all the data are connected through a common geospatial variable, it is possible to develop algorithms for a software system to automatically analyze the data for a possible correlation as soon as new data become available and mark the possible correlation for further analysis. The development of a geotagging system for real-time early warning for flooding in Vietnam was recently reported [12]. Also, a geotagging project in Thailand was completed to identify the distribution of dengue hemorrhagic fever cases with spatial information. The project was conducted with an aim to provide appropriate interventions focusing on controlling and preventing the disease and developing strategic planning [13].

In the United States, a group of public health researchers obtained about 80 million geotagged tweets from more than 600,000 nonduplicate Twitter users over a year period in order to determine the associations between Twitter indicators such as happiness, diet, substance use (alcohol, drug, smoking), physical activity, and the county-level health outcomes. The study drew conclusions for the probable associations between Twitter indicators of happiness, food, and physical activity and the decrease in premature mortality, obesity, and physical inactivity rate [14]. Tweets related to alcohol use also indicated a greater alcohol-use correlated mortality rate. The study indicated that real-time data collected from social media platforms can make it possible for public health experts to detect movement of norms, sentiment, and behaviors that may signify health emerging issues or outbreaks [14].

The recent outbreak of the coronavirus emphasizes the crucial role of MedicalGIS. An AI-based app was developed to predict whether a patient had been infected with the COVID-19 coronavirus by the sound of their voice, breathing, and coughing [20]. Even though this technology was expected to support the detection of emerging hotspots, its use of a camera to collect location information, voice, and sound carried the potential for data compromise. The app was reported to have a not very high accuracy rate of 70% [20]. Given the risk of data leaking and low accuracy rate, whether the benefits outweigh the risks is still under debate. Although experts claimed that thermal screening does not yet have the ability to detect the coronavirus, it was an effective method to screen Ebola patients at airports [21]. Recently, there was also an effort to employ deep learning and thermal-based cameras to detect coronavirus patients based on body temperature in public places [22]. GPS data can be mapped with these systems to recognize the patient’s location and determine the distribution of patients in a region. In the United States, Apple and Google developed a contact tracing app named COVID-19 Exposure Notifications to support health care agencies in tracking the places that an infected patient has been and the people with whom they have been in close proximity [23]. The US government also has been discussing methods of using smartphone location data to fight the coronavirus with technology companies and public health experts, including tracking whether people are maintaining appropriate social distancing [23]. However, preserving privacy policies presented barriers to the implementation of such tools. For other types of data, removing individual identifiers can be sufficient to protect people’s privacy. But that may not be the case with geotagging data.

Despite of the advantages and disadvantages, geotagging and geovisualization in health care are essential due to their abilities to support decision making, preventive care, and epidemiological efforts. The challenge lies in identifying situations where these solutions would be most effective and beneficial.

### Geotagging Best Practices: How to Get Started

To effectively use geotagging in health care, it is necessary to use the correct types of data (ie, base layers). The North American Association of Central Cancer Registries Inc created a geotagging best practice guide for the cancer registry community to ensure a high level of confidence, reliability, standardization, and accuracy in geotagging endeavors [24]. This guide outlines the components of geotagging with information to work effectively with geocodes at national, state, county, and city-block levels.

Another example of a best practice is the Digital National Spatial Data framework [25], which is meant to “assemble geographic information that describes the arrangement and attributes of features and phenomena on the Earth. The infrastructure includes the materials, technology, and people necessary to acquire, process, store, and distribute such information to meet a wide variety of needs” [26]. This framework is unique in that spatial data are not the only component; of equal importance are the people, organizations, and technology that make spatial analysis a national effort. The Digital National Spatial Data framework also proposes methods for disseminating geospatial data and illustrates the importance of funding geotagging projects.

Despite the motivation to create greater standardization and develop frameworks to assemble the information, there is an immediate need to understand the basic elements of geotagging and the data sources where the geocodes can be referenced. Therefore, the remainder of this paper will examine the necessary base layers of geotagging that can be adopted in health care and examine the sources of information that can assist in the implementation of MedicalGIS.

### Objectives

The objective of this paper is to consolidate basic knowledge to help medical professionals begin using geotagging in health care. This paper is designed as a resource document by suggesting the optimal geocodes, their sources, and a rationale for use. Geotagging limitations are also discussed.

## Methods

This paper consolidates the available geotagging resources (free and for fee) that can be leveraged by researchers and quality improvement personnel to effectively perform geospatial analyses primarily targeting the US health care and discusses the following layers and workflow of the geotagging framework:

1. Geotagging (first layer): translating of text-based descriptions to map coordinates and placing the data on the map
2. Geomapping (middle layer): finding the appropriate or available granularity level (city, county, zip code, and country) of the data you are working on and mapping/tagging the data to that geographic level
3. Adjustment and analysis: looking at the other data available for this geographic location and comparing this location to other locations with similar data and calculating necessary statistics
4. Conclusions and action: evaluating whether the outcome is good or bad, drawing conclusions, and recommending further research

The paper concludes with a summarization of tools that could be used for geotagging, including software applications that are either licensed or open-source platforms.

## Results

Information on the base geotagging layers that can be used to place a data point on a map is shown in Multimedia Appendix 1. The base layers are presented at various levels of granularity. At the most practical granular level is the ZIP+4 code, which can be used to present data at a geographic layer within a 5-digit zip code mapping segment. At the least granular level is the country, which presents data for each country in the world. The information is presented in order from the most granular to the least granular and includes a proposed preferred name for specific variables in future datasets.

Information on resources that can be used to adjust geocodes is shown in Multimedia Appendix 2. For instance, the intent of an analysis may be to assess the mortality rates for heart failure across all hospitals in the United States. In order to effectively plot this data a zip code alone will not suffice. The distinct hospitals with accompanying geocodes need to be known. Multimedia Appendix 2 offers resources that can be used for adjustment to effectively conduct a spatial analysis.

Information on data that can be used to evaluate outcomes based on a geographic location and adjustment data is shown in Multimedia Appendix 3. The data includes information such as health outcomes, financial information, prevalence of chronic diseases, and patient satisfaction. The intent of Multimedia Appendix 3 is to present a measured outcome at a specific geographic level (from Multimedia Appendix 1) with or without accompaniment of the adjustment data (from Multimedia Appendix 2).

Although the information in these tables is not an exhaustive list of resources that can be used to effectively adopt geospatial analysis in a health care setting, the resources are still comprehensive and applicable to commonly conducted analyses. These resources can be adopted to initiate an evaluation of hospital performance, patient demographics, health disparities, and the incidence of disease.

**Figure 1:**
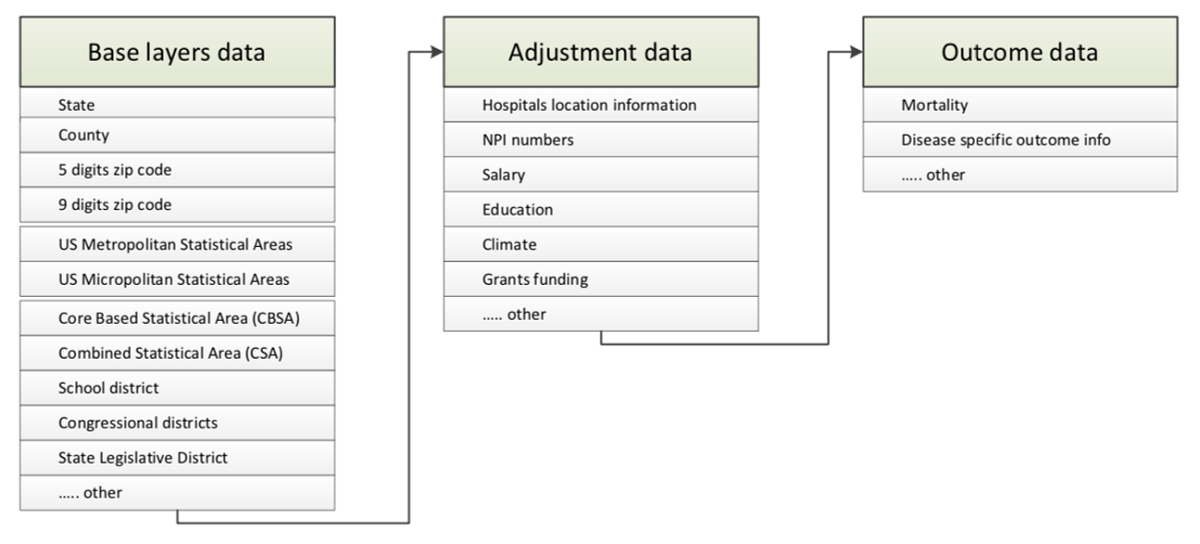
Proposed 3-layer model (green blocks) for using geocoded health care data with example data elements (gray blocks).

Figure 1 offers a graphical depiction of how the data from Multimedia Appendices 1-3 can be used in concert to conduct a geospatial analysis. Beginning with the base layer data presented in Multimedia Appendix 1, the data can be used to construct the boundaries for the geospatial analysis. The intent may be to represent data at the county, state, combined statistical area, or other applicable levels. Adjustment data shown in Multimedia Appendix 2 may be added to the analysis to segment the data by a specific category, such as hospital or provider, or based on socioeconomic variables. The outcome data in Multimedia Appendix 3 compose the final layer to a geospatial analysis and are used to generate the measure. These measures are often compared across the base layers and adjustment layers.

## Discussion

### Principal Findings

Despite the significant impact that geotagging can have in health care, there has been limited adoption. This is largely attributed to challenges associated with adopting software and acquiring data sources and a skills gap with implementation and interpretation. This paper summarizes the necessary data layers of geotagging that can be adopted in health care and examines the sources of information that can assist new users in the implementation and use of MedicalGIS.

Spatial analysis methods have expanded over the past half century resulting in an increased number of GIS software tools or updates to existing tools. In many instances software vendors are able to accommodate updates to the original software, while others have opted to provide access to third party applications for extensible use. Additionally, many frameworks offer programming languages, software development kits, and/or extraordinary interfaces for developing novel analytical tools or approaches.

There are numerous software applications that can be adopted for geospatial analysis. Many are commercial products and require a license for use while others are noncommercial products that are either open source or free. These tools are made available either as a web-based or locally installed application. These applications typically include functionalities to support rapid map generation, dynamic interaction and summaries, drill-down capabilities, cross-tabulations, and a widespread suite of graphical options including 2D and 3D analyses. The advantage of commercial products is the degree of support and training that is offered. There are typically ample user guides, white papers, and training offered by licensed software vendors. Noncommercial products typically have limited training opportunities, more verbose and technical user guides, and little technical support but may offer more extensibility and flexibility when working with data.

Some of the free tools, such as the R statistical programming language (R Foundation for Statistical Computing), require significant training on how to use a scripting language to generate a geographic analysis. Despite the steep learning curve, R is free to implement and offers incredible flexibility with analyses. Licensed software such as ArcView GIS (Esri) offers training and support that may assist with an easier transition to implement geotagging analysis. However, the licensed software may be cost prohibitive for some parties. There is also significant growth in the development of business intelligence software such as Tableau (Tableau Software LLC) and QlikView (QlikTech International AB) that offer dashboard views of data and can automate the geotagging process. These tools are particularly useful for organizational monitoring and decision making and require very little training. The choice between a commercial or noncommercial product is usually related to user experience, cost, and time demands of performing an analysis. To learn more about GIS software applications, the Centers for Disease Control and Prevention developed a list of common tools used for geospatial analysis [27].

### Limitations

Although positive impacts have been realized with geotagging, significant challenges still remain, data privacy being one. The Health Insurance Portability and Accountability Act of 1996 regulations restrict the use of patient data for research if there is a risk of potentially identifying a person. Therefore, there are limits to the level at which an analysis can be conducted, which is largely restricted to zip code rather than a specific address. However, there are existing limitations in obtaining accurate location data in a small area. As the recent coronavirus outbreak has put lives of millions of individuals worldwide at stake, it is understandable that many governments accepted the risk of data compromise by tracking people’s location data in efforts to contain the virus. In China, visitors to public buildings must scan QR codes using their smartphones so the app can monitor people’s movements/locations and later notify the users if they have been in close contact with COVID-19 patients. Telecom providers (through call histories) and technology companies (via smartphone apps) in England, Italy, Austria, Israel, Germany, and the United States are collecting or sharing either (1) personal location and use data or (2) anonymous and aggregated data to facilitate mapping and contact tracing [28]. The anonymous aggregated location data were used to map hot spots and determine movement patterns. However, there were arguments that location data does not become anonymous by simply deleting the identifiers; therefore, releasing it may lead to data compromise. Also, the collected data may not be representative as only about 67% of people worldwide have cell phones [28]. The individual location data were used instead to identify people who came into close contact with COVID-19 patients. Nonetheless, GPS/global navigation satellite system technology is practically accurate to within 16 ft. radius under clear sky [29] while the contact tracing would be more accurate if it can correctly obtain positioning data within 6 ft. The accuracy is also reduced near obstacles such as trees, bridges, or buildings [29], and COVID-19 has a higher infection rate inside buildings where human interactions mostly occur and the air is more compact. There is no doubt that geotagging is powerful, but its benefits should overcome the risks, and determining scenarios where it can outweigh the risks is always crucial.

Additionally, many of the external data sources are licensed and require a fee to obtain access. Information integration is often crucial for decision making. Multiple studies have been performed to detect areas or communities most vulnerable to COVID-19. As senior populations were considered at higher risk, Massachusetts Institute of Technology employed geotagged data analysis to determine if senior care facilities were lacking resources by integrating the bed availability data and safety data at assisted living, long-term care facilities, and nursing homes [30]. In an effort to prove that communities experiencing health care disparities are more susceptible to COVID-19, the *Los Angeles Times* created a map reflecting quality of care such as health care provider supply, insurance coverage, and rate of poverty [30]. The *New York Times* also generated a map presenting the number of people and the daily in and out frequency of captives in prisons all over the United States. The map suggested that nearly 20,000 people enter and leave prisons weekly, making the high risk of developing outbreaks from these centralized facilities clear [30].

There is also a skill gap in how to use geographic data and software effectively. Health care organizations typically do not have a GIS expert on staff, requiring them to purchase costly software or hire consultants to perform geographic analyses. While many GIS software applications can be costly and require training to use, there are free and open source software options that can be explored to minimize the cost. The need for training, however, is not often overcome.

Research also indicated that public health studies incorporating geocoded databases should also develop methods to evaluate and verify accuracy [31], as the error rate is often very high. Other issues are the cost of using this technology and hardware and software, training, and maintenance costs. GIS software is traditionally expensive. Although the cost of GIS hardware has decreased over time, it is still considered costly. GIS training is time-consuming and, therefore, expensive. As a result, software maintenance cost is still high.

Another challenge is to identify the required level of analysis for a geotagging project and select the appropriate base layer to support this requirement. For instance, a project that requires a block view of data may be difficult to implement given the challenges with acquiring data at this level. Commonly, the lowest level data is supplied at the 5-digit zip code. At this level, a regional analysis can be performed, but a more granular analysis (ie, block or address) is not possible. Also, frequent updates to 5-digit zip codes require organizations to maintain current sources. When the data intended to be geocoded is not current, this poses a challenge in establishing accurate geocodes.

Data quality is often a concern with geocodes. The quality of geographic data in health care is a continual challenge due to frequent updates to geocodes, lack of uniformity in their adoption, and inconsistencies in data collection parameters [32]. Not only are the geocodes themselves often limited and outdated, but the health care data sources, such as the public sources shown in Multimedia Appendix 3, should be subject to scrutiny. The data are often aggregated from other data sources or suppressed in areas with a low sample size. These limitations to the public data can result in a lack of complete coverage in geographic regions, particularly in rural areas with a lower population. The real value of public health care data comes from combining several sources. Unique identifiers for providers, hospitals, counties, states, and regions can be leveraged to combine data sources. For example, census data for each county can be combined with county health ranking data. A challenge with combining data sources is to resolve many-to-many relationships. For example, a zip code can be related to zero, one, or many core-based statistical areas (CBSA). That is, a single zip code may be mapped to a micropolitan and metropolitan area. Therefore, an analyst can only obtain an approximation of the rural/urban classification for any given zip code. Although such limitations exist, careful data handling can resolve many of these limitations by combining base layer geocodes. For instance, Topologically Integrated Geographic Encoding and Referencing Shapefiles published by the US Census can be used to obtain latitudinal and longitudinal boundaries for CBSAs. These boundaries can be compared with the latitudinal and longitudinal coordinates for a single hospital. If that hospital is found to fall within the boundaries of a CBSA, an analyst can classify the hospital as falling within a rural, micropolitan, or metropolitan area with greater confidence. Finally, there is inconsistent application of geotagging standards and best practices to identify the best data sources and software.

### Conclusion

This paper provides clear instructions and explanations on how geotagging can be leveraged in health care. It also offers the necessary initial resources to obtain geocodes, adjustment data, and health-related measures. Despite the motivation to move forward with geotagging in health care, there remain significant challenges around training and acquiring the appropriate software. Data integration, data security, and privacy are crucial and need to be addressed carefully. This technology can be very helpful; however, benefits and potential risks should be carefully identified before implementation. The resources outlined in this paper can support an individual and/or organization in initiating a geotagging health care project.

## Appendix

Multimedia Appendix 1 Base layers for geotagging health care data.

Multimedia Appendix 2 Adjustment data to limit the geocodes to specific categories (eg, hospitals, providers).

Multimedia Appendix 3 Public data sources that offer outcome measures for geotagging.

## Abbreviations

AI: artificial information
CDSA: core-based statistical areas
MedicalGIS: medical geographic information systems
