# Supplementary material for: Starter Kit for Geotagging and Geovisualization in Health Care: Resource Paper
Source: JMIR Form Res. 2020 Dec 24;4(12):e23379. doi: 10.2196/23379 (PMC7790608; doi:10.2196/23379)
Supplement: Multimedia Appendix 1 [file formative_v4i12e23379_app1.docx]

**Supplementary Table I. Base Layers for Geotagging Healthcare Data.**

| **Name of field** | **Proposed name** | **Description** | **Source** | **URL** |
| --- | --- | --- | --- | --- |
| ZIP+4 | 9ZIP | The five-digit code together with four additional digits, which is used to determine a geographic segment within the five-digit delivery area. | SAS GIS Library based on the 2006 Second Edition TIGER/Line files from the Census Bureau. | [http://support.sas.com/downloads/download.htm?did=104189#](http://support.sas.com/downloads/download.htm?did=104189) |
| ZCTA | 5ZIP | A ZCTA is a ZIP code tabulation area that includes a five-digit code | Census.gov 2010 TIGER/Line Shapefiles | <https://www.census.gov/geo/maps-data/data/tiger-line.html> |
| City | CITY | Consolidated city FIPS codes | Census.gov SUB-EST2014 Subcounty Population Estimate from 2014 | <http://www.census.gov/popest/data/cities/totals/2014/files/SUB-EST2014_ALL.csv> |
| School district | SCHDT | A database of data on population statistics for school districts in the United States. | 2014 SAIPE School District Estimates FTP Directory | <http://www.census.gov/did/www/saipe/data/schools/data/2014.html> |
| Metro Area | MDIV | Metropolitan divisions within a metropolitan statistical area that have a single core with the smallest population of 2.5 million. | Census.gov CSA-EST2015-alldata  Combined Statistical Area Population Estimates from 2015 | <http://www.census.gov/popest/data/metro/totals/2015/files/CSA-EST2015-alldata.csv> |
| Core Based Statistical Area (CBSA) | CBSA | Counti(es) have at least one core of more than 10,000 population, together with adjacent counties, which have a high degree of social and economic integration with the core as measured through commuting ties with the counties associated with the core. | Census.gov CSA-EST2015-alldata  Combined Statistical Area Population Estimates from 2015 | <http://www.census.gov/popest/data/metro/totals/2015/files/CSA-EST2015-alldata.csv> |
| Combined Statistical Area (CSA) | CSA | Combined Statistical Areas contain at least two adjacent CBSAs with significant employment interchange. | Census.gov CSA-EST2015-alldata  Combined Statistical Area Population Estimates from 2015 | <http://www.census.gov/popest/data/metro/totals/2015/files/CSA-EST2015-alldata.csv> |
| Rural-Urban Commuting Area Codes | RUCA | Rural-Urban Commuting Area Codes combine the standard Bureau of Census Urbanized Area and Urban Cluster definitions with work commuting information to characterize all of the nation's Census tracts regarding their rural and urban status and relationships. | WWAMI Rural Health Research Center | <http://depts.washington.edu/uwruca/> |
| County | COUNTY | It is a no-duplicate five-digit Federal Information Processing Standard (FIPS) counties or county equivalents code. The first two digits identify the FIPS state code and the last three digits refer to the county code. | Census.gov CO-EST2015-alldata County Population Estimate from 2015 | <http://www.census.gov/popest/data/counties/totals/2015/files/CO-EST2015-alldata.pdf> |
| Congressional districts | CONDT | An electoral constituency that nominates a single member of congress. | Census Bureau’s MAF/TIGER geographic database | <https://www.census.gov/geo/maps-data/data/cbf/cbf_cds.html> |
| State Legislative District | STLEGDT | A constituency that nominates members of the house of representatives. | Census Bureau’s MAF/TIGER geographic database | <https://www.census.gov/geo/maps-data/data/cbf/cbf_sld.html> |
| State | ST | The two digits code that uniquely identifies a state or U.S. possession/territory. | Census.gov CO-EST2015-alldata County Population Estimate from 2015 | <http://www.census.gov/popest/data/counties/totals/2015/files/CO-EST2015-alldata.pdf> |
| Country | COUNTRY | Countries can be geocoded using the ISO 3166-1 alpha-2 or alpha-3 numeric country codes | The Countries of the World (COW) database contains 71 distinct fields of data for the 249 countries, territories and dependencies recognized by the United Nations. | <http://opengeocode.org/download/cow.php> |
