# Supplementary material for: Starter Kit for Geotagging and Geovisualization in Health Care: Resource Paper
Source: JMIR Form Res. 2020 Dec 24;4(12):e23379. doi: 10.2196/23379 (PMC7790608; doi:10.2196/23379)
Supplement: Multimedia Appendix 2 [file formative_v4i12e23379_app2.docx]

**Supplementary Table II. Adjustment data to limit the geocodes to specific categories (e.g., hospitals, providers).**

| **Name of database** | **Description** | **Source** | **URL** |
| --- | --- | --- | --- |
| AHA Hospital Database | A licensed database of more than 6,400 hospitals surveyed from the annual AHA survey. | American Hospital Association | <http://www.ahadataviewer.com> |
| NPPES Provider Directory | A database of all the active and inactive national provider identification numbers. | CMS | <http://download.cms.gov/nppes/NPI_Files.html> |
| Census.gov | The census data can adjust geocodes by categories of socioeconomic status, education attainment, race, among others. | U.S. Census | <http://www.census.gov/data.html> |
| Climate Data Online | Worldwide historical weather and climate data including station history, location information, adjustment data of station location, temperature, precipitation, wind, radar data, and 30-year Climate Normals. | National Centers for Environmental Information | <http://www.ncdc.noaa.gov/cdo-web/> |
| Wage Data | Wage data by area and occupation for the nation, region, state, and metropolitan areas. | Bureau of Labor Statistics | <http://www.bls.gov/bls/blswage.htm> |
| Directory of Medical Schools | Directory of medical schools in the world with information about year the program was initiated, whether the program is active, and a link to the school’s website. | World Federation for Medical Education & Foundation for Advancement of International Medical Education and Research | <http://avicenna.ku.dk/> |
