# Supplementary material for: Starter Kit for Geotagging and Geovisualization in Health Care: Resource Paper
Source: JMIR Form Res. 2020 Dec 24;4(12):e23379. doi: 10.2196/23379 (PMC7790608; doi:10.2196/23379)
Supplement: Multimedia Appendix 3 [file formative_v4i12e23379_app3.docx]

**Supplementary Table III. Public Data Sources that Offer Outcome Measures for Geotagging.**

| **Name of database** | **Description** | **Source** | **URL** |
| --- | --- | --- | --- |
| Data.Medicare.gov | Includes data from the “compare” databases for hospital, nursing home, home health, dialysis, supplier and provider level quality and outcome data. | CMS | <https://data.medicare.gov> |
| HealthData.gov | A repository of federal, state, and city agency’s healthcare related datasets | HHS | <http://www.healthdata.gov> |
| Data.gov | Data, resources, and tools for research, web / mobile APP developing, and data visualizations. | U.S. General Services Administration | <https://www.data.gov> |
| Flex Monitoring | A database of critical access hospitals and state level data on financial and quality outcomes. | University of Minnesota | <http://www.flexmonitoring.org/data/> |
| Health Indicators Warehouse | A repository of national, state, county, and hospital level data on a variety of health topics such as demographics, chronic diseases, and health behaviors. | National Center for Health Statistics | <http://www.healthindicators.gov> |
| National Health Inventory Survey | Annual data from the U.S. National Health Interview Survey on healthcare related topics at an individual and household level. | University of Minnesota | <https://ihis.ipums.org/ihis/> |
| County Health Rankings | County level data on health behaviors, access to care, and mortality | Robert Wood Johnson Foundation and the University of Wisconsin Population Health Institute | <http://www.countyhealthrankings.org/rankings/data> |
| America Health Rankings | State level data on the strengths, challenges, and highlights of healthy behaviors and disease risk. | United Health Foundation | <http://www.americashealthrankings.org/states> |
| Community Health Measures | County level data on health behaviors, mortality, chronic disease incidence, and birth rates. | CDC | <http://wwwn.cdc.gov/communityhealth> |
| HEDIS | A tool to compare U.S. health plans against performance measures related to care and services. | NCQA | <http://www.ncqa.org/HEDISQualityMeasurement/HEDISMeasures.aspx> |
| Dartmouth Atlas of Healthcare | National, regional, hospital, and provider level data on Medicare reimbursement, quality and effective care measures, and demographics. | Dartmouth Atlas | <http://www.dartmouthatlas.org> |
| CMS Data Navigator | An application to access CMS datasets and resources easily. | CMS | <https://dnav.cms.gov> |
| CDC WONDER | Public health data on population statistics, environmental factors, mortality, and disease incidence. | CDC | <http://wonder.cdc.gov> |
| FLASHE | Survey data from parents and adolescents on family life, physical activity, health, and eating habits. | National Cancer Institute | <http://cancercontrol.cancer.gov/brp/hbrb/flashe.html> |
| NHANES | Survey data on adults and children in the U.S. on health and nutritional status. | CDC | <http://www.cdc.gov/nchs/nhanes/> |
| AHA Hospital Database | A licensed database of more than 6,400 hospitals surveyed from the annual AHA survey. | American Hospital Association | <http://www.ahadataviewer.com> |
| HSRR | A searchable database of datasets, instruments, and software from a variety of data sources. | National Library of Medicine | [http://www.hsrmethods.org/DataSources/HealthServicesAndSciences ResearchResources.aspx](http://www.hsrmethods.org/DataSources/HealthServicesAndSciences%20ResearchResources.aspx) |
| NCES | Searchable database of colleges, schools, and educational agencies. | U.S. Department of Education | <http://nces.ed.gov/datatools/> |
| MCD | The Medicare Coverage Database includes all National Coverage Determinations and Local Coverage Determinations. | CMS | <https://www.cms.gov/medicare-coverage-database/overview-and-quick-search.aspx> |
